# Progressive exacerbation of neurological symptoms in a patient with neurosyphilis following T9-T10 intradural space-occupying lesion resection: a case report

**DOI:** 10.3389/fimmu.2026.1729673

**Published:** 2026-01-20

**Authors:** Yongpeng Meng, Yan Bo, Hongtao Wei

**Affiliations:** 1 The Neurological Disease Center,The Second Hospital of Gansu Province, Lanzhou, Gansu, China; 2 The Neurological Disease Center,Affiliated Hospital of Northwest Minzu University, Lanzhou, Gansu, China; 3 The Department of Medicine, Northwest Minzu University, Lanzhou, Gansu, China

**Keywords:** antisyphilis therapy, case report, immunosuppression, neurosyphilis, penicillin G, reactivation, spinal surgery, *Treponema pallidum*

## Abstract

**Background:**

Neurosyphilis is a disease of the central nervous system (CNS) caused by Treponema pallidum (syphilis spirochete), which usually presents with a variety of nonspecific symptoms and is easily misdiagnosed. Although Treponema pallidum can remain latent after treatment, reactivation may occur in the presence of a compromised or immunosuppressed immune system. This article reports a case of reactivation of neurosyphilis after spinal surgery, with the aim of exploring the possible role of immunosuppression in the process.

**Case presentation:**

The patient was a 75-year-old female admitted to the hospital with persistent bilateral lower extremity pain and weakness and a past history of syphilis that was cured 31 years ago. Upon admission, the patient was diagnosed with an intradural occupying lesion and underwent T9-T10 intradural space-occupying lesion resection. Postoperatively, the patient developed symptoms of neurogenic bladder and paraplegia, and further examination revealed abnormal cerebrospinal fluid suggestive of reactivation of neurosyphilis. Through syphilis antibody testing and cerebrospinal fluid analysis, the diagnosis of neurosyphilis was finally confirmed and antisyphilis treatment was started.

**Treatment and outcome:**

The patient received a 14-day course of intravenous benzylpenicillin, which resulted in significant symptomatic improvement and restoration of most functions at discharge. After 12 months of follow-up, the patient had fully recovered, with a progressive decrease in syphilis antibody titers and no neurosyphilis reactivation.

**Conclusions:**

This case emphasizes the importance of preoperative syphilis antibody screening, especially in elderly patients with a history of syphilis. The immunosuppressed state may contribute to the reactivation of syphilis spirochetes; therefore, it is important to consider this potential risk and take appropriate precautions when performing surgical treatments such as spinal surgery. Penicillin remains the therapeutic agent of choice for neurosyphilis, and early diagnosis and treatment are critical to the patient’s prognosis.

## 1 Introduction

Neurosyphilis is a disease of the central nervous system caused by *Treponema pallidum* infection. This disease is often considered a “mimic” in the extant literature because of its diverse clinical presentation and lack of specific symptoms. Today, syphilis infections caused by this spirochete have become a global public health problem (1). Neurosyphilis often poses a significant clinical management challenge for clinicians who lack experience in treating the disease (2). The disease often affects middle-aged and older populations, especially after prior syphilis cure, and reactivation may occur in the context of a compromised immune system or immunosuppressed state. Early clinical decision-making is crucial to improve patient prognosis, especially in areas where medical resources are scarce. Interestingly, we report a case of progressive exacerbation of neurologic symptoms in a 75-year-old female patient with neurosyphilis who underwent T9-T10 intradural space-occupying lesion resection. We propose a viewpoint on clinical decision-making regarding neurosyphilis that is not mentioned in the extant literature: if a patient suffers from both neurosyphilis and an intravertebral space-occupying lesion, surgical treatment of the intravertebral space-occupying lesion first may induce reactivation of syphilitic spirochetes, thus aggravating the neurosyphilis condition.

With this case report, we hope not only to improve clinical knowledge of neurosyphilis, but also to provide lessons for the management of similar patients, especially in balancing surgical treatment with infection control in complex clinical situations.

## 2 Methods

### 2.1 Design

A case report design was used in this study to document in detail the diagnostic process, treatment plan and clinical decision making of the patients. This was in reference to Zhuang et al.’s approach to writing case reports (3). All study steps followed the requirements of the Case Reporting (CARE) Guidelines (4). To ensure the legitimacy and transparency of the study, all data and materials were subjected to appropriate ethical review and strict privacy measures were taken.

### 2.2 Data management and security

In medically underserved and resource-inequitable regions, the overarching goal of clinical practice is to address as many patient needs as possible using limited resources while minimizing medical financial burdens. This demand is shared by the vast majority of patients facing economic hardship, and our report aligns precisely with this objective. We added descriptions of medical resource inequity or scarcity in the background section to better clarify the study’s positioning.

The disease addressed in this case report is *Treponema pallidum* infection, a pathogen that cannot survive in common mammals such as dogs and rabbits. As a result, animal studies to elucidate the immune and physiological mechanisms of *Treponema pallidum* are not feasible, and every human infected case thus becomes invaluable research data that advances our understanding of the disease.

To ensure the integrity and security of the study data, this study followed the data security management program established by Yifei Chen (5). Patient’s personal information and clinical data were encrypted to guarantee the transparency and compliance of the study process. If researchers wish to access our data for meta-analysis in the future, they may submit research proposals to us after obtaining ethical approval. This approach can maximize the utilization of human data on *Treponema pallidum*, which is critical because knowledge about the pathogen can only be derived from a limited number of human patients due to the lack of viable animal models.

### 2.3 Ethics

This is a human case report conducted with institutional ethical approval and written informed consent, consistent with CARE guidelines.

This case study was reviewed and approved by the Ethics Committee of Northwest Minzu University Affiliated Hospital (IRB: No. GSSEY2025-KY001-18). The patient signed an informed consent form authorizing the use of their personal information, imaging data, and follow-up data for the study.

## 3 Results

### 3.1 Patient information

**Figure 1:**
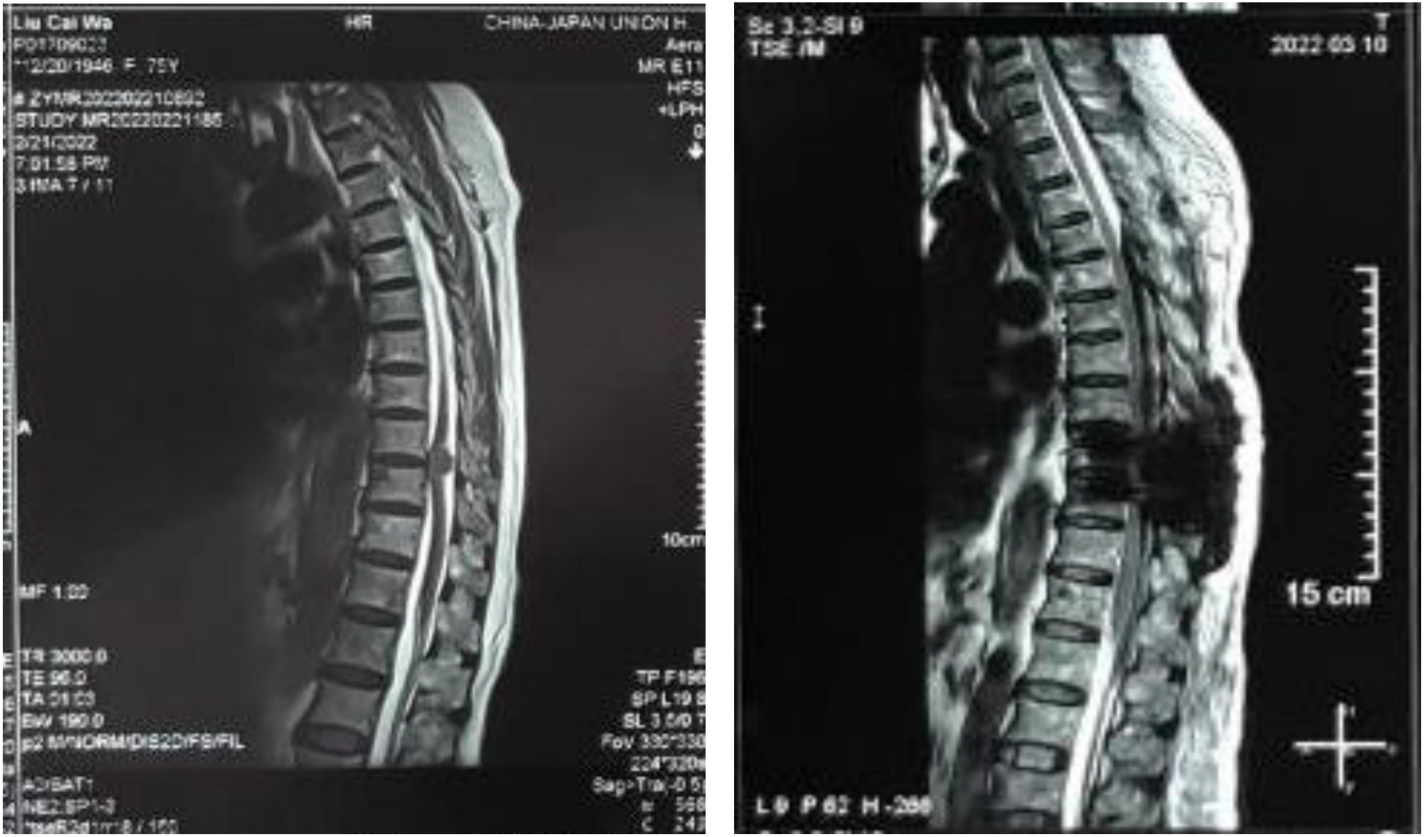
Thoracic lumbar spine MRI scan findings. The figure shows an intraspinal space-occupying lesion at the level of the patient’s T9-T10 intervertebral disc, which was confirmed by imaging as the source of the spinal lesion. The image was a preoperative finding that showed the exact location of the lesion and provided an important basis for the subsequent surgical plan.

A 75-year-old female patient felt bilateral lower back and leg pain lasting for about 1 year and felt no strength in bilateral lower limbs for the last three weeks. In order to relieve her symptoms, the patient came to the orthopedic outpatient clinic. The outpatient physician considered the patient’s condition to be severe and subsequently requested that the patient be hospitalized. The outpatient doctor ran a physical examination for the patient, which showed that the patient was conscious, had muscle strength of grade 4 in the left lower extremity and grade 3 in the right lower extremity. A magnetic resonance imaging (MRI) scan of the thoracic lumbar spine showed the presence of an intravertebral space-occupying lesion at the level of the 9–10 disc of the thoracic spine (**Figure 1**). Laboratory tests showed the patient to be positive for syphilis-specific antibodies; however, the patient stated that she was past cured of syphilis infection 31 years ago and provided no other information.

**Figure 2:**
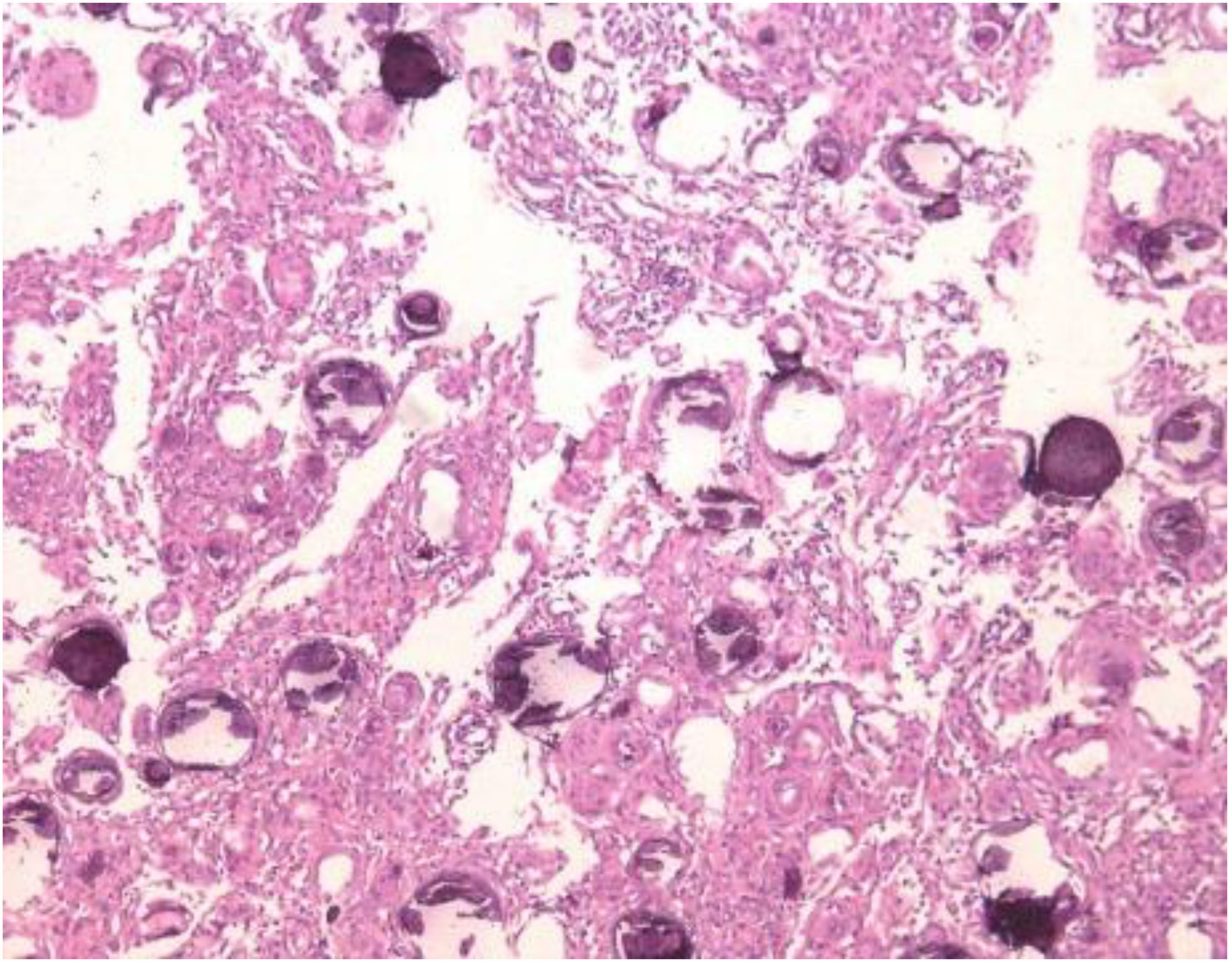
Postoperative pathologic findings. The surgically resected intradural occupying lesion was pathologically examined and shown to be a granulomatous meningioma of the sandy granuloma type.

As soon as the patient was admitted to the hospital (Day 0), the orthopedic inpatient physicians began to develop a surgical plan to address the patient’s intravertebral space-occupying lesion. On the 6th day of the patient’s hospitalization, the patient underwent T9–10 intradural occupying lesion resection and T9–11 vertebral body fixation surgery under general anesthesia. The intradural lesion removed during the procedure was pathologically examined and shown to be a granulomatous meningioma (**Figure 2**). On day 8 of the patient’s hospitalization, the patient felt that the muscle strength of both lower extremities had returned to at least level 4, however, the patient developed neurogenic bladder (abdominal distension, bloating, and diminished bowel sounds) and paraparesis (urinary and fecal incontinence, and a decrease in the muscle strength of both lower extremities to level 0) without any warning. These particular symptoms puzzled the orthopedic inpatient physicians, and although bleeding during surgery and compression of the spinal cord may present similar symptoms, the patient’s thoracic lumbar MRI scan showed no specific signals in the surgical area of the spinal cord (**Figure 1**). The orthopedic inpatient physicians thought the patient might have a neurologic condition and recommended transfer to neurosurgery for treatment.

**Figure 3:**
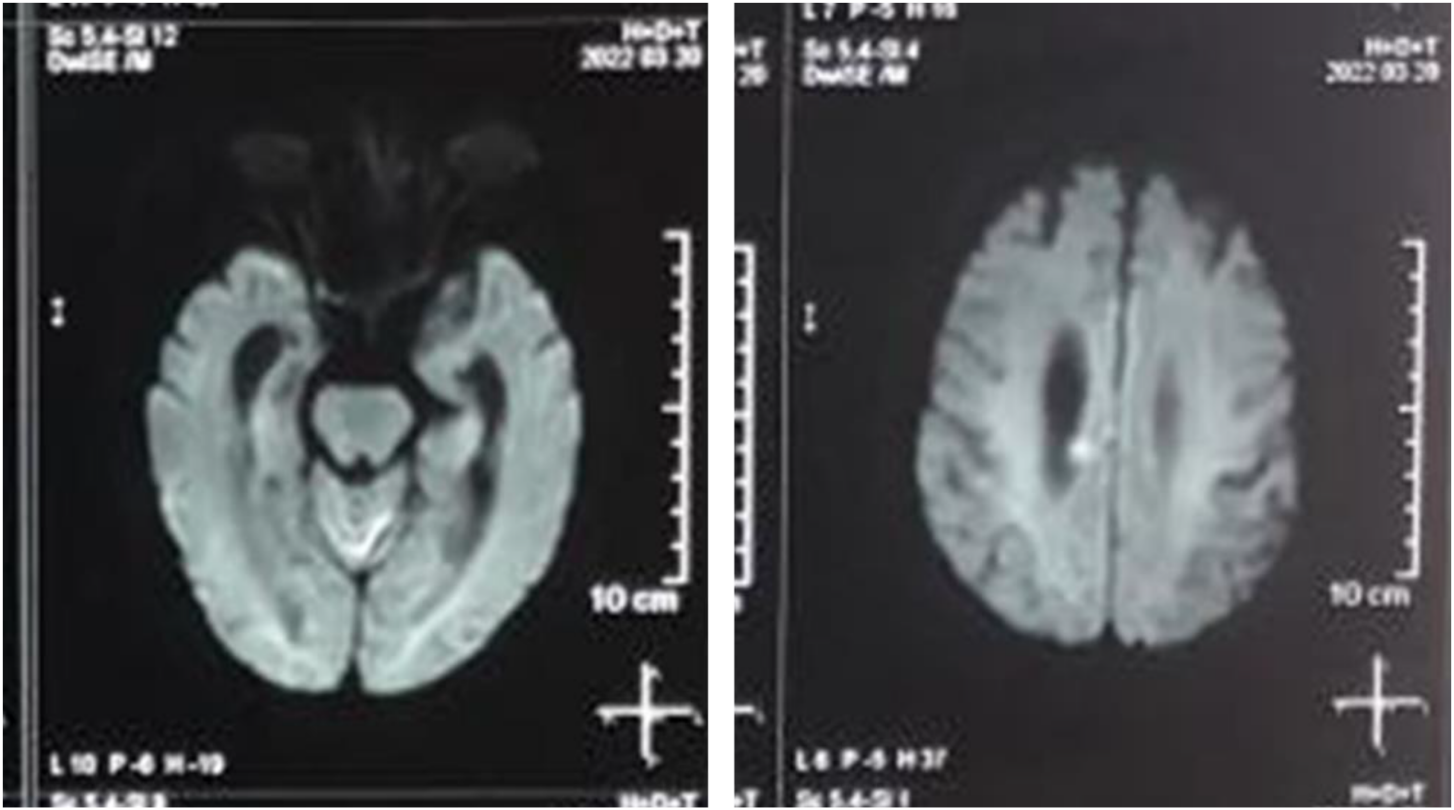
Results of cranial MRI scan. The patient underwent a cranial MRI on postoperative day 12, which showed scattered new cerebral infarcts in the right frontal lobe, right lateral ventricular apex, and left cerebellar hemisphere. Obvious areas of ischemia can be seen in the figure, suggesting possible CNS infection or inadequate perfusion.

On the 9th day of hospitalization, the Neurosurgery received this particular patient. Clinician at the Neurosurgery ran a physical examination on the patient, which showed that the patient was experiencing organic brain dysfunction accompanied by fever (maximum temperature of 38.5 °C), which was characterized by apathy, hunger strike, intermittent irritability, and even some neuropsychiatric syndromes. The clinician of the Neurosurgery tried to give an exploratory treatment with gastrointestinal decompression, intravenous nutrition, and antibiotic therapy. The results of the exploratory treatment showed that the patient’s temperature returned to normal on the 12th day of hospitalization, but the symptoms of neurogenic bladder worsened. To explore the cause, the patient underwent a cranial MRI scan, which showed scattered new cerebral infarcts in the right frontal lobe, right lateral ventricular apex, and left cerebellar hemisphere (**Figure 3**). The clinician of the Neurosurgery hypothesized that this was an acute cerebral infarction, as a CNS infection induced during or after surgery may present similarly. Based on this idea, the clinician of the Neurosurgery, performed a lumbar puncture on the 20th day of hospitalization. The pressure during the lumbar puncture was 210 mmH_2_O, and the cerebrospinal fluid (CSF) extracted from it showed an orange color (**Table 1**). The laboratory examination of the cerebrospinal fluid specimen showed a white blood cell count of 108×10^6^/L, cerebrospinal fluid protein of 1217.15 mg/L, glucose of 5.79 mmol/L, and chloride of 122.20 mmol/L. The clinician of the Neurosurgery gave the patient antibiotics of meropenem combined with vancomycin according to the laboratory examination of the cerebrospinal fluid. However, the patient’s major symptoms did not radically improve in the following month. On the 36th day, a lumbar puncture was performed to review the laboratory parameters of the patient’s cerebrospinal fluid specimen and found that the pressure was 120 mmH_2_O, the cerebrospinal fluid was translucent and colorless, and the leukocyte count was 2 × 10^6^/L. The cerebrospinal fluid protein was 379.60 mg/L, the glucose was 3.51 mmol/L, and the chloride ion was 120.40 mmol/L. From the data, the patient seemed to be getting better, but the patient was still suffering from the disease, and the patient’s symptoms did not radically improve. suffering from the disease, it was clear that the exploratory program of antibiotics with meropenem combined with vancomycin was a failure.

**Table 1:** Cerebrospinal fluid findings of the patient.

Time	Cerebrospinal fluid protein (mg/L)	Glucose (mmol/L)	Chloride (mmol/L)	Leukocytes (×10^6^/L)	Transparency	Color
Normal reference ranges	150-450	2.5-4.4	120-132	0-5	Clear	Colorless
Day 21	1217.15	5.79	122.20	108	Turbid	Orange
Day 25	532.7	4.66	121.70	3	Clear	Yellowish
Day 39	379.60	3.51	120.40	2	Clear	Colorless
Day 43	288.4	4.01	122.0	2	Clear	Colorless

This table displays the patient’s CSF indices at multiple time points during hospitalization, with normal reference ranges included for comparison. Initial results suggested a potential infectious lesion; subsequent improvements in CSF indices reflect clinical progress.

### 3.2 Clinical findings

On the 41st day of admission, the clinician of the Neurosurgery incidentally discovered that the patient’s syphilis-specific antibodies were positive, so it was likely that the patient was experiencing a syphilis infection. The clinician of the Neurosurgery reviewed the patient’s past medical records and considered this clinical hypothesis for two reasons. First, at the time of admission, the patient only stated that the patient had been diagnosed and adequately cured of syphilis 31 years earlier and did not discuss the course of treatment or subsequent follow-up. Second, the topic of syphilis as a “mimic” did not come to the attention of the resident during the course of this admission when he discovered that the patient was positive for syphilis-specific antibodies. The previous intravertebral space-occupying lesion (**Figures 1**, **2**) did seem to require more urgent treatment than the syphilis infection in terms of the risk of disease progression. For these two reasons, there is insufficient basis to exclude the patient from syphilis infection.

### 3.3 Diagnostic assessment

**Figure 4:**
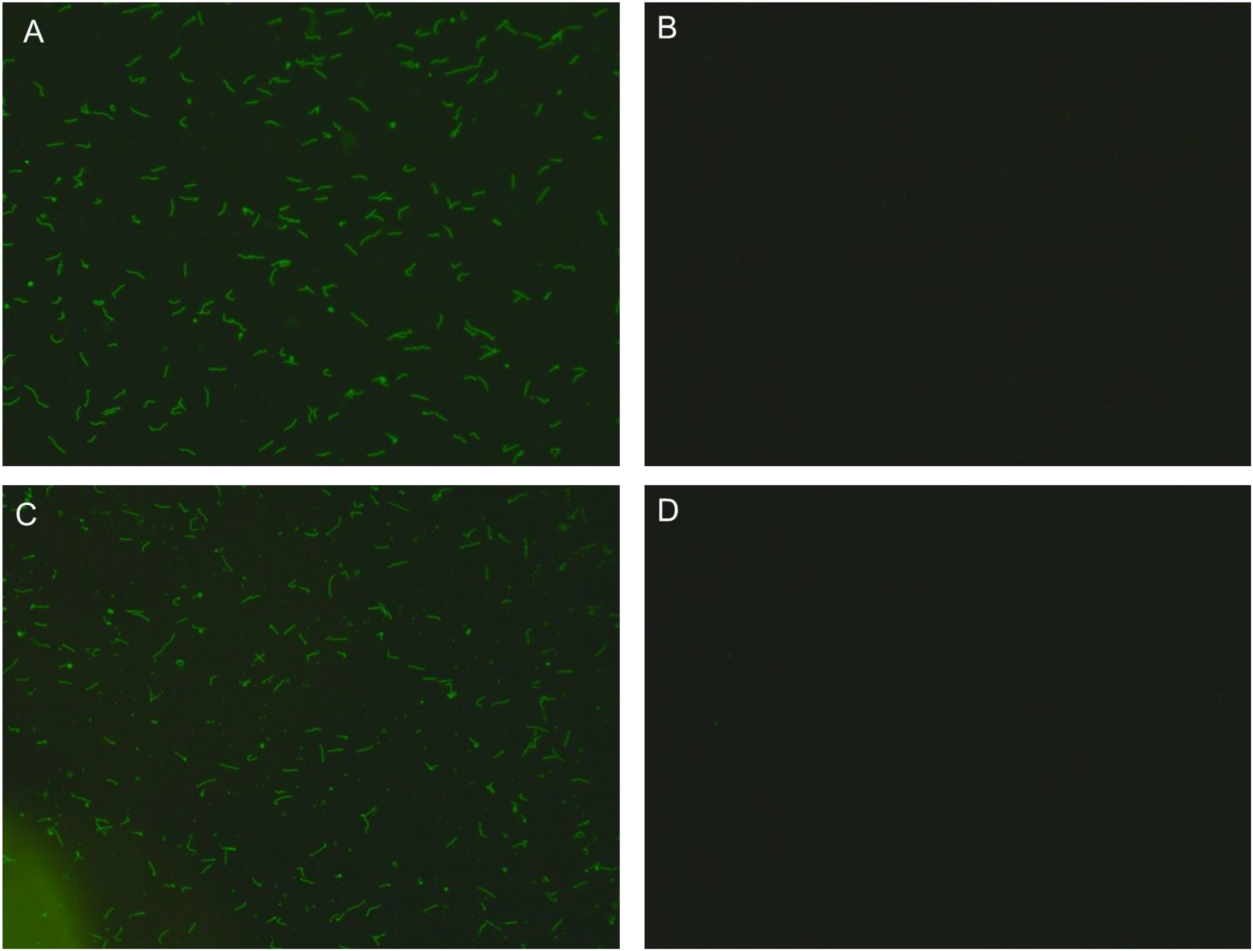
Results of syphilis spirochete antibody tests. Results of IgG and IgM antibodies to syphilis spirochetes in cerebrospinal fluid and serum. The figure shows the patient’s syphilis spirochete antibody test, which includes IgG and IgM antibody responses to the cerebrospinal fluid specimen, as well as IgG and IgM responses to the serum specimen.Positive IgG antibody results support the diagnosis of neurosyphilis. **(A)** IgG antibody to spirochetes of syphilis in a cerebrospinal fluid specimen; **(B)** IgM antibody to spirochetes of syphilis in a cerebrospinal fluid specimen; **(C)** IgG antibody to spirochetes of syphilis in a serum specimen; **(D)** IgM antibody to spirochetes of syphilis in a serum specimen.

The clinician of the Neurosurgery invited an Infectious Disease clinician for a clinical consultation as soon as he learned of clinical hypothesis. The Infectious Diseases physician recommended testing the patient for syphilis antibodies. The patient’s serum and cerebrospinal fluid specimens (lumbar puncture on day 41) were then tested for syphilis antibodies and found to be positive (**Table 2**). The lumbar puncture was evaluated with a pressure of 140 mmH_2_O and the cerebrospinal fluid appeared translucent and colorless. Laboratory examination of the cerebrospinal fluid specimen revealed a white blood cell count of 2 × 10^6^/L. Cerebrospinal fluid protein was 288.4 mg/L, glucose was 4.01 mmol/L, and chloride was 122.0 mmol/L. At this point, we proposed a diagnosis of neurosyphilis based on (1) the patient’s serologic test for syphilis antibodies was positive, and (2) the patient’s current presence of neurologic signs and symptoms, such as an appearance of apathy, hunger strike, intermittent irritability, and neuropsychiatric syndrome; and (3) the cerebrospinal fluid specimen was positive for syphilis antibodies. We reviewed with interest the 2020 European Guidelines for the Management of Syphilis, which recommend that any situation in which evidence of syphilis antibodies fails to reject a diagnosis of syphilis infection should result in an immediate and arbitrary diagnosis of syphilis infection (6). Current evidence confirms the clinical hypothesis of neurosyphilis (**Figures 3**, **4**).

**Table 2:** Syphilis antibody dynamics.

Time	RPR	TPPA	CSF-TPPA	Clinical significance
Day 0	N.A.	Positive	N.A.	Past treated infection
Day 26	1:8	Positive	Positive	Detected neurosyphilis; reactivation
Day 55	1:2	Positive	Positive	Treatment effective
Day 146	1:1	Positive	Positive	Treatment effective
Day 238	Negative	Positive	Positive	Past treated infection; serofast

Demonstrates the dynamic changes of syphilis antibodies in patients at different time points, including the results of RPR, TPPA and CSF-TPPA tests. The table records the changes in antibody titers before and after the patient’s treatment, reflecting the effectiveness of the treatment and the reactivation of syphilis spirochetes.

### 3.4 Therapeutic intervention

Based on this new clue, on day 43 of admission, we instituted intravenous benzylpenicillin at 18 million IU for a duration of 14 days.

### 3.5 Follow-up and outcomes

Based on this new clue, on the 43rd day of admission, the patient experienced significant clinical relief from antisyphilis treatment and gradually began to actively eat and drink. The patient reported that his legs were stronger (muscle strength level 3) compared to his previous stay in the orthopedic inpatient unit. The patient completed 14 days of high dose benzylpenicillin IV therapy with significant symptomatic improvement and was discharged on day 57 of hospitalization.

In order to closely monitor the patient’s condition after discharge, the clinician of the Neurosurgery began 12 months of daily follow-up visits by phone. The daily follow-ups consisted of asking about existing symptoms and urging the patient to have his serum anti-syphilis antibodies rechecked every 3 months. At the third month follow-up visit, the patient revealed that his serum anti-syphilis IgG had dropped to 1:2. After one year of follow-up, the patient had fully recovered (RPR: negative; TPPA: positive; **Table 3**).

A retest of serum anti-syphilis antibody IgG 1:2 was positive. The patient’s clinical symptoms basically disappeared, his speech was clear, his questions and answers were relevant, his diet and defecation were normal, the muscle strength of both lower limbs was grade 4, Bartholin’s sign was negative, and he could take care of himself. After 1 year of follow-up, the patient is now completely self-care and can do daily housework.

### 3.6 Timeline

The timeline of the patient’s clinical course described above is presented in **Table 3**.

**Table 3:** Clinical symptoms and treatment schedule.

Time	Event	Interventions
Day 0	Admission, MRI showed T9-T10 occupancy	Preoperative preparation
Day 6	T9-T10 occupancy resection	Surgery
Day 8	Muscle strength of both lower limbs recovered to grade 4	–
Day 13	Neurogenic intestinal paralysis and paraparesis developed	Transferred to neurosurgery
Day 20	Lumbar puncture, abnormal cerebrospinal fluid	Started meropenem combined with vancomycin
Day 43	Anti-syphilis therapy started (benzylpenicillin for 14 days)	Gradual improvement with symptomatic relief
Day 57	Discharged	Follow-up planned for one year

Demonstrates the patient’s clinical symptoms and therapeutic measures during hospitalization, including date of surgery, symptomatic changes, treatment options, and clinical response. The timeline helps to clearly demonstrate the progression of the patient’s condition and the timeliness of treatment.

## 4 Discussion

### 4.1 Literature review

Yan Bo and Yifei Chen concluded that reviewing the literature in the PubMed database related to the clinical hypotheses proposed in the studies could improve the credibility of the new findings (7). We followed the search steps of previous systematic reviews by Fei Zhao’s research team (8), utilizing “case” and “neurosyphilis” as keyword searches.

This case suggests mandatory pre-surgical screening for RPR/TPPA in patients with previous syphilis infection, even if claimed to be cured. If positive, lumbar puncture is required to rule out neurosyphilis, otherwise surgical stress may induce reactivation of the infection.

In this case, we have new information that syphilis infection can reactivate at specific times even after treatment. Small amounts of residual syphilis spirochetes may remain after adequate treatment. These residual syphilis spirochetes go dormant, slowing down their metabolism and minimizing exposure to the penicillin-binding target, the key enzyme for peptidoglycan synthesis. The main purpose of peptidoglycan synthesis by S. syphilis is to synthesize its own cell wall (9). Therefore, when the spirochete enters a hypometabolic state, penicillin, which inhibits cell wall synthesis as a mechanism of bacterial inhibition, has a very limited effect on the spirochete, but clinical evidence for this claim is currently lacking (10). Syphilis spirochetes also evade immune surveillance by adsorbing fibronectin from the human host while they are in a dormant state. If the immune function of the human host is out of balance and the syphilis spirochetes enter a reproductive phase that benefits them more than the dormant phase, they will begin to multiply again, causing targeted damage to the body. Currently, no effective treatment exists for the complete elimination of dormant syphilis spirochetes (10).

The optimal approach is to screen regularly for syphilis serologic tests Rapid Plasma Reactivity (RPR) and Treponema pallidum Particle Agglutination (TPPA), and cerebrospinal fluid specimens should also be tested in the presence of neurologic symptoms in a regimen somewhat similar to that proposed by the laddered test of Czulada et al. (1).

Previous reports in the literature have suggested that a patient’s nonspecific antibodies can be converted from positive to negative to indicate an inactive syphilitic infection; whereas patients with specific antibodies show lifelong positivity (2, 11). It has also been reported that only if the time point of detection of syphilis infection is within the window of elimination of the serofixation period, it is possible to convert all specific and nonspecific antibodies to negative after adequate treatment (2). However, the patient in this case was negative for IgM in both serum and cerebrospinal fluid, and IgG was positive in both serum and cerebrospinal fluid on the premise that he was cured of syphilis 31 years ago, which suggests that relying only on the results of the antibody screen to determine the time point of syphilis infection is inaccurate. Based on information obtained from the literature, we propose a theoretical hypothesis.

### 4.2 Theoretical hypothesis

Two reviews since 2025 have revealed the correlation between changes in patients’ immune status and neurosyphilis reactivation (12, 13). Varun Sethi and Michael Marks examined critical reports on previous clinical guidelines, diagnosis, and treatment of neurosyphilis, and proposed that global scholars put forward more referenceable and bold theoretical hypotheses from a non-penicillin treatment-oriented perspective (14). For instance, Madhusudan Dalvi retrospectively analyzed the fever therapy for syphilis infection proposed by Julius Wagner-Jauregg in the past from a contemporary perspective. At that time, this bold theoretical hypothesis was audaciously verified in some voluntary syphilis patients. The underlying principle was to raise body temperature to weaken the viability of Treponema pallidum. This discovery led Julius Wagner-Jauregg to be awarded the Nobel Prize in Physiology or Medicine in 1927 (15). Even though we now have penicillin and have thus forgotten fever therapy, this does not mean that we cannot propose bold and speculative theoretical hypotheses.

From the lessons learned from this acquisition of clinical prioritization decisions, we must put clinical decisions for syphilis infections first. If the lesion or symptom that we consider important is treated first, then this will deplete the immune reserve capacity within the patient’s organism. This idea was reported by Yan Bo in previous international conferences on the distribution pattern of immune information (16, 17). In other words, if a routine blood report shows an extremely elevated white blood cell count, then this is an indication of a depletion of the host’s reserve of immune cells for the immune system to respond to the pathogen. Imagine assuming that the current immune reserve capacity of the entire body is deployed to the brain to deal with the immune response to infection after surgery, wouldn’t those immune messages to monitor the syphilis spirochete be understocked? This would stimulate the syphilis spirochete to shift from dormancy to reproduction. This pattern of distribution of immune information is usually accompanied by an inflammatory state centered on platelets, and Yan Bo implies that close attention to platelets could be a breakthrough in monitoring and treating inflammatory symptoms (18). A study of surgical patients showed a significant reduction in the expression of CD3^+^ T cells and the co-stimulatory molecule CD28 after surgery, while regulatory T cells (Tregs) were not suppressed, suggesting that T cells mediate immunosuppressive effects after surgical trauma and bleeding (19). Another study in patients undergoing surgery for lung cancer showed that surgical trauma significantly suppressed T cell function through the programmed death-1 (PD-1)/programmed death ligand-1 (PD-L1) pathway. The number of T-lymphocytes decreased after surgery, and anti-PD-1 antibodies partially reversed T-cell apoptosis. These imply that surgical trauma can induce immunosuppression through the PD-1/PD-L1 pathway (20). Two lines of evidence suggest that the immunosuppressed state is a trigger for reactivation after herpes zoster infection (21, 22). In addition, one strong piece of evidence suggests that patients with syphilitic spirochete RPR conversion turn positive for RPR, i.e., syphilitic spirochete reactivation, during immunosuppressive therapy (23).

This theoretical hypothesis was proposed against a backdrop of bold speculation. There is no validatable dataset in our case data to support its verification. This means that in the future, we can conduct appropriate animal experiments to test the effect of inhibiting platelet activity or enhancing immune reserve in controlling neuroinflammation and neurosyphilis, an approach that may serve as an exploratory direction for the treatment of neurosyphilis (24).

### 4.3 Primary “take-away” lessons

Syphilis infection is a legacy of the last century. Those patients who were successfully treated with antisyphilis therapy in the last century are now elderly. Based on the principles of pathologic damage and immune defense of the syphilis spirochete (9), a subset of special patients may have their own immune or nutritional functions that cause the still-remaining syphilis spirochete to switch from the reproductive to the dormant phase. When such specialized patients are exposed to specific events, such as aging and surgical procedures, the syphilis spirochetes may be prompted to switch from the dormant to the reproductive phase. This can lead to multiple, mutually unexplained symptoms that are not effectively relieved by symptomatic treatment.

The first clinician must be alert to the patient’s syphilis antibody screening results. A diagnosis of syphilis infection should be made arbitrarily once the patient has any condition that does not allow for a ruling out of syphilis infection.

### 4.4 Patient perspective

The patient said: “The disease had been nagging me for quite some time and my quality of life had plummeted. I was always inexplicably upset during my hospitalization until I received penicillin treatment. I felt my pain disappeared after the first penicillin treatment. I am very grateful to The clinician of the Neurosurgery, who insisted on calling me every day to check on my condition for 1 year after I was discharged from the hospital.”

### 4.5 Limitation

Reinfection as a potential cause merits consideration, given the patient’s report of syphilis treatment 31 years prior. A key limitation of this case report is the inability to definitively rule out reinfection as the etiology of the patient’s neurosyphilis, as opposed to reactivation of latent infection. Epidemiologically, reinfection is less probable due to the patient’s age and remote history of treated syphilis, yet it cannot be entirely dismissed. The absence of IgM antibodies in both serum and is more consistent with reactivation, as primary infections typically elicit an IgM response. However, definitive differentiation between these two scenarios would require molecular strain typing of Treponema pallidum from CSF. Thus, while the temporal association with surgery strongly suggests reactivation, coincidental reinfection cannot be excluded.

Another critical consideration is the lack of direct pathogen identification. The conclusion hinges on the premise of *Treponema pallidum* reactivation, yet there is no mention of PCR or other direct detection methods for the spirochete in CSF or tissue. While clinical practice often relies on serological findings and CSF profiles for diagnosis, discussion of the absence of PCR data is essential for methodological completeness, especially in light of the marked CSF protein elevation and pleocytosis observed on Day 20.

We operate in a medically underserved region, where such direct pathogen detection approaches (including PCR for T. pallidum in blood or CSF samples, and electron microscopic examination of CSF specimens) are more precise but prohibitively expensive. Given that the diagnosis could be inferred via antibody kinetics, we opted not to impose additional financial burdens on the patient, who was confirmed to have economic hardships. This practical constraint, rooted in regional resource scarcity and patient affordability, accounts for the lack of direct pathogen detection data.

## 5 Conclusion

With the rising incidence of syphilis globally, there has been an increase in the number of cases of latently infected elderly patients undergoing surgery. The experience in this case suggests that although intravertebral space-occupying lesions seem to deserve higher clinical priority, unnecessary hospitalization would be reduced if syphilis antibody screening results were given high priority by the initial clinician (57 Days vs. 14 Days). Routine preoperative syphilis antibody screening for elderly patients undergoing surgery should be prioritized in clinical practice. This measure can expedite differential diagnosis of neurosyphilis, shorten hospital stays, and reduce medical resource waste. The “mimicry” of the clinical presentation of *Treponema pallidum* infection can make early clinical decision-making difficult for inexperienced physicians, and implementation of the 2020 European Guidelines for the Management of Syphilis is highly recommended.

## Data Availability

The original contributions presented in the study are included in the article/supplementary material. Further inquiries can be directed to the corresponding author.
